# OntoCheck: verifying ontology naming conventions and metadata completeness in Protégé 4

**DOI:** 10.1186/2041-1480-3-S2-S4

**Published:** 2012-09-21

**Authors:** Daniel Schober, Ilinca Tudose, Vojtech Svatek, Martin Boeker

**Affiliations:** 1 Institute of Medical Biometry and Medical Informatics (IMBI), University Medical Center, 79104 Freiburg, Germany; 2 University of Economics, Prague, Nám. W. Churchilla 4, 130 67 Praha 3, Czech Republic

## Abstract

**Background:**

Although policy providers have outlined minimal metadata guidelines and naming conventions, ontologies of today still display inter- and intra-ontology heterogeneities in class labelling schemes and metadata completeness. This fact is at least partially due to missing or inappropriate tools. Software support can ease this situation and contribute to overall ontology consistency and quality by helping to enforce such conventions.

**Objective:**

We provide a plugin for the Protégé Ontology editor to allow for easy checks on compliance towards ontology naming conventions and metadata completeness, as well as curation in case of found violations.

**Implementation:**

In a requirement analysis, derived from a prior standardization approach carried out within the OBO Foundry, we investigate the needed capabilities for software tools to check, curate and maintain class naming conventions. A Protégé tab plugin was implemented accordingly using the Protégé 4.1 libraries. The plugin was tested on six different ontologies. Based on these test results, the plugin could be refined, also by the integration of new functionalities.

**Results:**

The new Protégé plugin, OntoCheck, allows for ontology tests to be carried out on OWL ontologies. In particular the OntoCheck plugin helps to clean up an ontology with regard to lexical heterogeneity, i.e. enforcing naming conventions and metadata completeness, meeting most of the requirements outlined for such a tool. Found test violations can be corrected to foster consistency in entity naming and meta-annotation within an artefact. Once specified, check constraints like name patterns can be stored and exchanged for later re-use. Here we describe a first version of the software, illustrate its capabilities and use within running ontology development efforts and briefly outline improvements resulting from its application. Further, we discuss OntoChecks capabilities in the context of related tools and highlight potential future expansions.

**Conclusions:**

The OntoCheck plugin facilitates labelling error detection and curation, contributing to lexical quality assurance in OWL ontologies. Ultimately, we hope this Protégé extension will ease ontology alignments as well as lexical post-processing of annotated data and hence can increase overall secondary data usage by humans and computers.

## Background

With the advent of the semantic web and RDF-based knowledge representation techniques off-the-shelf ontology editors like Protégé 4 [1] gain widespread use. Although its functionality is sufficient for daily ontology editing tasks, some pre-release clean-up checks on the ontology, especially in the area of class naming conventions and metadata availability, can complement Protégé 4 in a useful way. It was shown that inconsistencies in **naming conventions **can impair readability and navigability of ontology class hierarchies, and even hinder their alignment and integration [2]. An initial specification for typographic, syntactic and semantic naming conventions for life science ontologies [3] has been introduced by the OBO Foundry [4]. It was shown that clear naming conventions for editor-preferred class names (e.g. stored in the rdfs:label or rdf:ID/OWLClassName) provide guidance to ontology creators and help developers avoid flaws and lexical inaccuracies [2] when editing, but especially when interlinking ontologies. By increasing the robustness and exportability of ontology class labels, adherence to explicit class naming conventions can foster communication when ontology engineers need to collaborate with external groups to align their ontologies and facilitate the import and usage of classes from external ontologies or imported ontology modules. Naming conventions increase the robustness of context-based text mining for automatic term recognition and text annotation and they ease the manual and automated integration of terminological artifacts, i.e. comparison, orthogonality-checking, alignment and mapping. Robust labeling generally eases the access to ontologies through meta-tools such as provided by the NCBO BioPortal [5], i.e. by reducing the diversity with which these tools have to deal, thus reducing the burden on tool and ontology developers alike. Ultimately, following clear labeling guidelines can facilitate ontology re-use and reduce redundant development.

Another area that can profit from tool support is **metadata enrichment**: Although 'expensive' to add, metadata stored along a class in self-defined annotation properties or standardized elements provided by metadata policy providers like Dublin Core [6] will ease the human understanding of the editorial, administrative and semantic nature of ontologic entities. Before a new ontology version is released for public use, it should be checked if all metadata elements that are mandatory within a particular design principle documentation, e.g. annotation properties like natural language definitions or class labels, are present in the ontology and the ontology is hence assumed to be sufficiently described for the human user.

Based on own previous experience, we think the actual status of metadata completeness and labelling consistency can be improved, especially where lack of compliance is due to missing software capabilities. This need for tool support is also exemplified by pre-release tests implemented independently within different groups to check on metadata availability and labelling consistency, e.g. as seen in the OBI project [7] and in the Disease Ontology project [8] respectively.

To assist ontology editors in complying with metadata requirements and naming conventions outlined in their style guides and design principle documentations, we here introduce a Protégé plugin that checks an OWL ontology loaded into Protégé against naming conventions and metadata completeness specified by the user. Specifically, our plugin intends to contribute to lexical harmonization by validating class names according to specified checks. We here present the OntoCheck plugin, which intends to ensure naming consistency by testing for defined label patterns and allows for amendments in the area of metadata analysis.

## Implementation

The OntoCheck plugin was implemented as a plugin for the Protégé 4.1 ontology editor using the Protégé OWL API (version 3.2.2) and Java version 1.6.0_22. An informal requirement analysis was conducted on the basis of the OBO Foundry naming conventions [3] and on-going editing work in the different ontology engineering projects the authors were involved in. To test and to quantify OntoChecks capabilities, as well as gather further requirements, we applied the plugin within different projects and investigated the following six ontologies: Biotop [9], DCO [10], NTDO [11], GoodRelations [12], Vehicle Sales Ontology [13], and @neurist ontology [14]. For each, we created, stored and applied a different set of checks.

## Results

The OntoCheck plugin is available for download at our website (http://www.imbi.uni-freiburg.de/ontology/OntoCheck/).

The list of identified high level software requirements, together with an indication on fulfillment and implementation by the plugin is presented in Table 1.

**Table 1:** Requirements for a naming convention and metadata verification tool

Requirement	Aspects met and Implementation	OntoCheck Panel
Easy installation, usage and intuitive navigation.	Protégé plugin, structured into 3 self-explaining tabs. Tooltips providing on-the-spot guidance.	All
Generation and display of numeric counts for selectable ontology metrices.	Making use of the Protégé and Java API, diverse metrices are available, amending the already present 'Ontology Metrics'.	All
Selection of an 'entry class node' from where on - leaf-wards - a check should be done.	Allows to test for a certain postfix e.g. '_Disposition' only within a selected 'Disposition' entry node sub-tree. Allows checking for metadata availability in selectable subtrees.	All
Display of classes failing a specified test and export as list.	Found classes can be sorted according to different criteria and exported for later curation.	All
Display of quantitative results on detected issues in terms of absolute and percentage counts in a given subtree.	A statistical data pane verbalizes the numerical results in a copyable natural language sentence.	All
Storage and reload capabilities for created checks allowing for later re-use and propagation.	An xml file is generated storing all checks in a reproducible way.	All
Detection for 'presence' and 'required cardinality' of labels and metadata.	Checks are available on OWL elements capturing lexical information, i.e. rdf:ID, rdfs:label, own annotation properties and standard annotation properties e.g. from Dublin Core or SKOS.	Check
Check for syntactical and typographical patterns and label length i.e. to discover too short or too long names within string values of selectable entities.	Allows checking naming conventions via simple string matches and full regular expressions. Checks the length of labels. A significant fraction of the OBO Foundry naming conventions can be checked, i.e. case, separator but also morphemic conventions.	Check
Detection and counts of redundant class labels.	Label repetition can be checked for via the ComparePanel.	Compare
Comparison of values between pairs of entities to detect similarities and avoid redundancies.	Operators like equals, contains or starts with can be used to compare selectable entities.	Compare
Quantification of ontology measures useful for ontology evaluation, progress monitoring and complexity analysis.	Displays the percentage or absolute number of entities having 'exactly', 'at least' or 'at most' a certain number of annotation properties, direct sub-/superclasses, or 'usages', i.e. indicating 'hub nodes'.	Count

The high level requirements are listed in the first column followed by their specific implementations, indicating the extend of requirement fulfilment in our tool. The last column indicates in which tab the function is implemented.

### OntoCheck functionalities

#### Testing for cardinalities and metadata completeness

**Figure 1:**
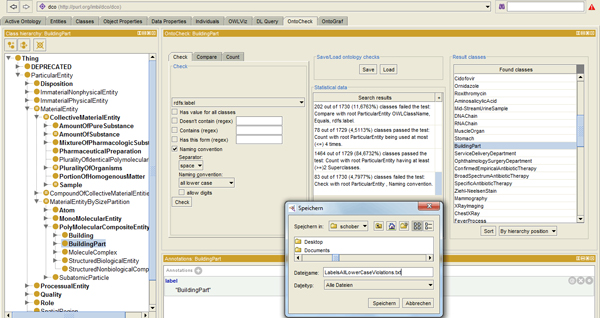
**The Check panel**. The Check panel displays the specification (left) of a test for an 'all lower case, space separator' naming convention on rdfs:label for the active ontology. The 'statistical data' view (middle) lists a history of launched checks and quantifies their results in terms of absolute amount of classes failing a test. Percentages are given with respect to the overall number of entry node descendants. One of the result classes, 'BuildingPart', is activated to show the found violation in the label, which is MixedCase as seen in the metadata pane below. Clicking on a class in the result pane (right) will activate it in the class hierarchy pane (left) opening a metadata edit pane to allow for corrections (below). The lower right corner shows how a file name and location can be selected to export the result list.

In the Check panel (Figure 1) a user can **select any entity **provided in an active open ontology as default, imported or self-defined annotation metadata. Lexical examples are rdfs:label (default), dc:comment (imported), and definition (self-defined). All entities can be **checked for presence **of values, e.g. the user can specify that all classes should have at least a label or a natural language definition. Classes lacking the specified metadata are displayed and can be amended accordingly.

#### Testing for lexical patterns in names with regular expressions

The ability to correlate an entity with standard Java regular expression as listed for java.util.regex.Pattern [15] can be used to check names for the presence or absence of specific lexical **prefix, infix or postfix patterns**. E.g. a regular expression of the form .*ValueRegion|.*Region can be used to test for **explicitness in labels**, .i.e. all 'ValueRegion' subclass names should contain either the explicit postfix 'ValueRegion' or 'Region'. This function also allows to detect **'metalevel' postfixes **like '_class', '_type', '_concept', or '_relation'. Also **stop-words **like 'A' and 'the', as well as **Boolean operators **('and', 'or'), and lexical indications for **negations **('non', 'anti 'or 'dis') can be detected and abolished from names.

Checks for minimum and maximum **character and word count **can identify potentially unclear names, e.g. being shorter than 4 characters or unreadable names longer than e.g. 50 characters or 10 words. Checks for punctuation, e.g. if dots are present, allow for the **detection of abbreviations**, while all-upper-case-checks can detect **acronyms**. Checks for **cardinality indicators **within names could be used in a semantic analysis guiding expressivity selection, e.g. words indicating cardinality requirements, such as 'minimal', 'maximal' might hint for the selected OWL EL profile not to be sufficient.

#### Testing for typographic naming conventions

The Check panel allows verifying whether a particular naming convention is fulfilled for a chosen entity, e.g. if all values for the rdf:ID/OWLClassName in a selected subtree comply to an 'all-lower-case-underscore-separator' convention. We here list the **typographic and syntactic checks **possible:

**Word Separator**: An entity can be checked for none, space, hyphen, underscore and dot separator conventions.

**Word Case**: An entity can be checked for all lower case, ALL UPPER CASE, Upper case start, camel Hump and Camel Case conventions.

**Digits**: An entity can be checked for numbers in labels, e.g. to look for cardinality and order indicators.

#### Comparing values between specified entities

**Figure 2:**
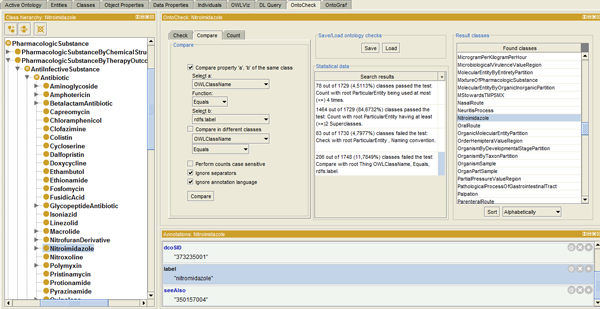
**The OntoCheck Compare panel**. The Compare panel displaying a check that verifies whether the rather dynamic rdfs:label still matches a previously given static semantic ID (OWLClassName), given word separators are ignored. A considerable amount of classes is found, i.e. the detected class 'Nitroimidazole' (marked), which have deviant labels, i.e. here 'Nitromidazole' (without "i" after "Nitro", see annotation metadata below).

The Compare panel (Figure 2) allows **comparing the values of annotation properties and metadata within a class or between different classes**. For example for each class in a subtree it can be checked if the rdf:ID matches the rdfs:label of the same class using the equals, contains or starts with operator. Case and separator and language awareness can be adjusted. As a result, classes with different values for the specified entity are listed, and can now be rectified, i.e. for the mentioned case, avoiding divergence in meanings between labels. Furthermore, a **check for label redundancy and naming clashes **in equal (synonymous) fields for different classes helps avoiding hidden redundancies, e.g. alerting on classes with the same name in a different namespaces.

#### Quantifying ontology measures for ontology evaluation

**Figure 3:**
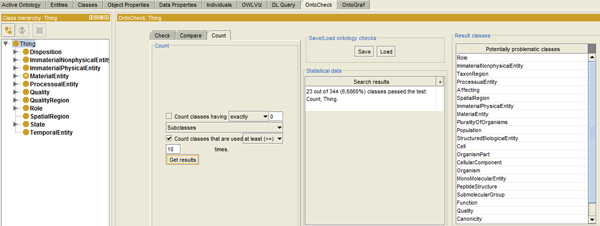
**The OntoCheck Count panel**. A count for 'hub-node' classes is carried out over the whole Biotop ontology (entry node is Thing). A list of 23 classes used more than 10 times is displayed in the Result classes' pane.

The Count panel (Figure 3) detects and **quantifies ontology measures for ontology progress monitoring, evaluation and complexity analysis**. Specifically, it displays the percentage and absolute number of nodes having exactly, at least or at most a certain number of selectable metadata elements, parents and children, direct super- and subclasses, as well as class usages. For example, counting the annotation properties per class allows to detect classes having >= 1 assignments for one and the same label type, e.g. definition, versionInfo or term ID, which should not be allowed. Counting direct sub-/superclasses can be used as proxy measure for 'how much is known' (for the asserted case) and 'how much can be inferred' (for the post-reasoned case). Too many immediate subclasses may indicate overly flat structures. Counting descendants/ancestors indicates the 'ontological depth' or 'relative specificity/granularity' in terms of 'root-distance' respectively.

Counting the 'usages' of a class, e.g. listing all classes with no 'usages' in restrictions other than subclassing named classes allows detecting 'ontological isolates' that have no dependencies. As such orphans are ignored by other logical definitions; they could potentially be removed or hidden in a simplified view of an ontology, focusing on the ontologies' defined and embedded classes linked via object properties. Analyzing the amount of richly axiomatized classes, - so-called 'hub nodes' - helps to determine how much work was put into an ontologies computer accessible semantics. Listing hub nodes that have many in- and outgoing relations also provides a proxy for the core domain described in the ontology, as these are likely to represent the more important classes in a formalized domain. As an application is likely to focus on these 'key classes', particular care must be taken to ensure that domain coverage is of sufficient granularity here.

### The OntoCheck user interface

The OntoCheck plugin provides a new editing tab within Protégé and is organized into the **three subpanels **Check, Compare and Count (see Figures 1, 2, 3), being largely **self-explainable **and easy to understand and use. **Tooltips **are displayed for most items upon 'mouse-over object' actions. Each tab shows the class hierarchy pane to let a user select an entry node and the annotations pane in order to make the amendments as required by the test results. Each pane allows specifying the check pattern in the left half and provides the test results in the right half of the pane.

All specified **check constraints are stored in a 'history list' and can also be stored in an autogenerated external XML file**, as an editor is likely to do the same check on an ontology repeatedly, i.e. as pre-release check. The stored check-specification file can also be exchanged and shared among a group of developers. All **result classes can be sorted **alphabetically or according to hierarchy position. Result lists can subsequently be enriched with the lacking metadata; either directly or they can be **exported as txt file and distributed **among curators for later or concurrent curation.

The main tab for curating naming issues is the first panel opened per default, the Check panel. The Compare panel allows comparing the values for specified entities and the Count panel allows measuring how often a class is used in formal definitions. Additional screenshots can be found on the OntoCheck website.

### Testing the OntoCheck tool

To detect software errors and test the tool against its requirements, six ontologies were checked with the tool. Each author tested two ontologies from different engineering efforts, covering a wide thematic scope, from the biomedical domain over the educational domain, up to the business domain. Overall over sixty single checks have been carried out, half of the checks tackling naming conventions, the other half metadata completeness and metrics counts. For only three checks a specific entry class was selected as target node (checked subtree): In order to check for standard affixes to keep the label explicit, the subtrees Role, ValueRegion and Disposition were selected. Table 2 illustrates where the selected ontologies could be improved by applying OntoCheck and quantifies the found violations. For a more detailed study we refer to the upcoming ICBO 2012 conference proceedings [16].

**Table 2:** Exemplary OntoCheck tests with quantification of detected violations

Ontology	Entry Node	Entity	Panel	Check	Classes [abs, %]
BioTop	root	<rdfs:label>	Check	Upper case start	12 (4)
BioTop	root	<owl:Class rdf:about>	Check	CamelCase	34 (8)
DCO	root	<ru-meta:definition>	Check	Min card.=1	37 (8)
DCO	'Disease'	<SNOMED_ID>	Check	Min card.=1	2 (2)
DCO	root	<ru-meta:synonym>	Count	Min card.>2	238 (40)
DCO	root	<ru-meta:shortLabel>	Check	Max Char Count < 20	3 (.5)
DCO	root	n/a	Count	CountClsHavingAtLeast15Subclasses	15 (1)
DCO	root	n/a	Count	CountClsUsedAtLeast15times	48 (3.3)
NTDO	root	<rdfs:label>	Check	Doesn'tContain'Class'or'class'	3 (1)
Good Relations	root	<rdfs:label>	Check	Min card.=1	6 (15)
Vertical Sales Ontology	root	<rdfs:label>	Check	Length regex.{4,50}+	1 (1.5)
Vertical Sales Ontology	root	<rdf:ID>	Check	Doesn'tContain'Or'	7 (10)
Vertical Sales Ontology	root	n/a	Count	ClsUsedOnlyOnce	13 (20)
@neurist	root	n/a	Count	CountClsHavingExactlyOneSubclass	150 (5.3)

'Entry Node' refers to the selected class in the hierarchy for which all descendants are tested. The entity selected to be checked is described via its OWL syntax element. The last column indicates the amount of found classes violating (Check panel) or fulfilling (Count panel) a specified pattern. For the naming checks 'abs' refers to the absolute count of entities of the specified type failing the test. '%' refers to the ratio of abs to the amount of all entry node descendants.

## Discussion

The usefulness of ontology design principles in general, and naming conventions in particular, increases considerably when supported by ontology editing tools. This had been shown earlier, e.g. for the Kismeta Validator [17], which was developed under a related paradigm, but focused on XML schemata and DB labels.

Looking at the practical application scenarios with examples outlined in the result section, we see that the OntoCheck plugin meets most of the desired specifications. It helped in discovering and alleviating labeling errors, fostered metadata enrichment and allowed to investigate an ontologies formal expressivity. Specifically, the plugin allows for word case and delimiter checks, regular expression matching (affix checks), cardinality and entity comparison checks.

Of the sixteen OBO Foundry naming conventions [3] six could be checked with our plugin (nearly 40%) [16]. The remaining conventions, that OntoCheck was not able to check for, would rely on a thorough lexical analysis requiring a lexicon, which is not yet implemented in this version of the plugin. However this could be amended by integrating the LiLa framework for 'linguistic analysis of entity labels in ontologies' [18], providing an interface to various natural language processing tools and resources for deeper terminological analysis.

Rendering labels in ontologies more consistent will pave the way for tools that use lexical information in class names for ontology integration, formalization and inconsistency detection, e.g. like OBOL [19], which recommends logical definitions for new classes and cross-products by exploiting lexical information from labels. Discussions have started in the OBO domain, where OORT, the OBO Ontology Release Tool is currently being developed [20] to include such label checks into their release tool. OntoCheck would make a useful addition to this tool, given its functionality would be delivered as a standalone Java library using solely the OWL API, rather than using the Protégé API.

Lexical ontology alignment tools such as the PROMPT tool suite [21] will be served with more robust information making automatic alignment and integration easier and more reliable. Recently, ontology alignment and transformation techniques have been designed that explicitly rely on naming structures over the ontology graph [22], and thus will particularly benefit from a prior clean-up.

As long as accepted recommendations for certain combinations of single naming conventions are not available, we can only enable checks on a *per-convention *basis, rather than allowing multiple checks simultaneously, e.g. defined in overall naming convention sets, e.g. the Foundry vs. Manchester vs. Stanford style convention sets. If naming conventions were accessible in a standardized repository, one could envision checks and enforcements of whole naming schemes to be drawn from such libraries. In this regard, we have joined forces with the ontology design pattern community [23] to transform naming conventions into formal reusable Naming ODPs. We also investigate the reimplementation of parts of OntoCheck as a webservice in order to foster integration into Semantic Web portals like Watson [24], which would ease reuse for portal and library providers, as semantic metrics can be updated continuously and used for ontology comparison, evaluation, ranking, e.g. helping to select compatible artefacts with similar design principles to be aligned or merged easily.

At the moment the user has to amend violating labels manually, but for many cases names violating tests could be corrected (semi-)automatically in an 'OntoCure-mode' in the future. For an extensive and updated list of desired and upcoming features, please visit the OntoCheck webpages.

## Conclusions

Although in an early development stage, the OntoCheck plugin proved already useful in carrying out pre-release checks for ontologies in different projects [9-14]. It has helped alerting developers on labelling violations and contributed in keeping these ontologies clean from naming errors. It also rendered the ontologies more complete by curing the lack of metadata. Carried out as pre-release check, the OntoCheck tests contributed to quality assurance [25] in the mentioned projects. Ultimately, we hope this Protégé extension will contribute to secondary data usage by rendering class names more robust and consistent, hence easing lexical post-processing of annotated data.

## Availability and requirements

**Project name**: The OntoCheck Plugin

**Project home page: **http://www.imbi.uni-freiburg.de/ontology/OntoCheck/

**Operating system: **Platform independent

**Programming language: **Java

**Other requirements: **Java 1.5.1 or higher, Protégé 4.1 or higher

**License: **GNU GPL

## Competing interests

The authors declare that they have no competing interests.

## Authors' contributions

DS initiated and supervised the project and wrote the initial paper draft. IT implemented the OntoCheck plugin and contributed ideas. VS revised the manuscript and contributed ideas. MB gave additional comments and revised the manuscript. DS, MB and VS were involved in testing the tool and commit software error reports to IT.
